# A Brain-Inspired Model of Theory of Mind

**DOI:** 10.3389/fnbot.2020.00060

**Published:** 2020-08-28

**Authors:** Yi Zeng, Yuxuan Zhao, Tielin Zhang, Dongcheng Zhao, Feifei Zhao, Enmeng Lu

**Affiliations:** ^1^Research Center for Brain-Inspired Intelligence, Institute of Automation, Chinese Academy of Sciences, Beijing, China; ^2^Center for Excellence in Brain Science and Intelligence Technology, Chinese Academy of Sciences, Beijing, China; ^3^National Laboratory of Pattern Recognition, Institute of Automation, Chinese Academy of Sciences, Beijing, China; ^4^School of Artificial Intelligence, University of Chinese Academy of Sciences, Beijing, China

**Keywords:** theory of mind, false-belief task, brain inspired model, self-experience, connection maturation, inhibitory control

## Abstract

Theory of mind (ToM) is the ability to attribute mental states to oneself and others, and to understand that others have beliefs that are different from one's own. Although functional neuroimaging techniques have been widely used to establish the neural correlates implicated in ToM, the specific mechanisms are still not clear. We make our efforts to integrate and adopt existing biological findings of ToM, bridging the gap through computational modeling, to build a brain-inspired computational model for ToM. We propose a Brain-inspired Model of Theory of Mind (Brain-ToM model), and the model is applied to a humanoid robot to challenge the false belief tasks, two classical tasks designed to understand the mechanisms of ToM from Cognitive Psychology. With this model, the robot can learn to understand object permanence and visual access from self-experience, then uses these learned experience to reason about other's belief. We computationally validated that the self-experience, maturation of correlate brain areas (e.g., calculation capability) and their connections (e.g., inhibitory control) are essential for ToM, and they have shown their influences on the performance of the participant robot in false-belief task. The theoretic modeling and experimental validations indicate that the model is biologically plausible, and computationally feasible as a foundation for robot theory of mind.

## 1. Introduction

Theory of Mind (ToM) is the ability to infer and understand other people's mental states to predict their behavior (Premack and Woodruff, 1978). It is a fundamental cognitive ability for the social brain. One of the most critical milestones in the ToM development is gaining the ability to attribute false belief: that is, to recognize that others can have beliefs about the world that are diverging (Wimmer and Perner, 1983). There is a wide variety of false-belief task (Huang and Liu, 2017; Scott and Baillargeon, 2017), but most of them can be divided into unexpected transfer task (Wimmer and Perner, 1983), unexpected contents task (Perner et al., 1987), and appearance-reality distinction (Flavell et al., 1983). Flavell et al. (1983) present a classical unexpected transfer task, Sally-Anne Test: Sally first placed a marble into her basket; then, she left the scene, and the marble was transferred by Anne and hidden in her box. Then Sally returned, and children were asked a belief question “Where will Sally look for her marble?” If the children pointed to the previous location of the marble, it meant that the children could understand that Sally held a false belief about the marble's location. Most 4-year-olds could point to the correct location, but most 3-year-olds failed—they predict that Sally will find her marble in the box.

As indicated in Asakura and Inui (2016), although ToM research has made progress on empirical findings and theoretical advances, relatively few efforts have been made from the biological plausible computational models' perspective, especially for false belief understanding. Based on findings of neural correlates and mechanisms of the false-belief task, we propose and build a Brain-inspired model of Theory of Mind (Brain-ToM model). And we challenge the false-belief task by incorporating the proposed model to humanoid robots. In this paper, we only focus on non-verbal unexpected transfer tasks as described below, including how to learn to understand object permanence and visual access from self-experience and use them to infer other's belief and predict their behavior. The object permanence is the ability to understand that objects continue existence even it cannot be perceived (Piaget and Cook, 1952).

From our point of view, self-experience in autobiographical memory and its utilization to infer other's belief or predict other's action is fundamental and crucial to the ToM. It is also mentioned as self-projection in Buckner and Carroll (2007) or using memories to understand others (Moreau et al., 2013). It enables real understanding of the self and others as well as their relationships, and utilize them to infer others' mental states based on personal experience from the self point of view. This perspective seems somewhat missing in existing research about the computational model.

In our opinion, an agent who can infer other's belief and predict their behavior should have the capability of self-other distinction as the premise. So in Zeng et al. (2016, 2017), we proposed a brain-inspired robot bodily self-model with the neural mechanisms of bodily self-perception based on extensions to primate mirror neuron system, and apply it to the humanoid robot for self-recognition. In this paper, based on the related findings for neural correlates and mechanisms of the ToM, we propose a Brain-ToM model to make the humanoid robot learn from self-experience. With the Brain-ToM model, the robot can pass the non-verbal unexpected transfer tasks adapted from Senju et al. (2011) and Southgate et al. (2007). The efforts may also provide a possible computational model and hints on how infant infers and understands other people's beliefs. Compared to the previous model, the characteristics of our model are with relatively more solid details from the biological brain. It explores the effect of self-experience as a core and is with considerations on the maturation of correlated brain areas (e.g., calculation capability) and their connections (e.g., inhibitory control). Besides, the model is naturally a brain-inspired spiking neural network model and is fundamentally based on brain plasticity principles.

The rest of this paper is organized as follows: Section 2 reviews the related work of computational models, the false belief tasks, and the brain regions in the ToM. In section 3, the architecture of the Brain-ToM model, the concrete neural network architecture, the Voltage-driven Plasticity centric Spiking Neural Networks (VPSNN), and the inhibitory control mechanism are introduced. The experimental settings, the experimental results and analyses are given in section 4. Some discussions and conclusions are drawn in sections 5, 6, respectively.

## 2. Related Works

In this section, we briefly review several related works, including the computational models, the false belief tasks, and the related brain regions of ToM.

### 2.1. Computational Models

Berthiaume et al. (2013) presented a constructivist connectionist model to simulate the false-belief task. The model encoded the location of an object, whether an agent has observed the object's movement, and the location where the agent came back to search. With the increased hidden units to improve computational power, the model would predict the correct search in two different false belief tasks—the approach task and the avoidance task. Their model was the first computational model to autonomously construct and transit between structures and to cover the two major false-belief task transitions. They suggested the view that the source of the transition is not developed in the understanding of beliefs, but changes in auxiliary skills such as: executive function, understanding and using representations, working memory, or language. Goodman et al. (2006) built two Bayesian models named CT model (copy theorist) and PT model (perspective theorist). Beliefs were only correlated to the location of the toy in the former model, and in the later model, the belief was not only correlated to the toy's location but also Sally's visual access, i.e., could Sally saw the toy moved or not. With the increase of resources and complexity in the PT model, the model could pass the false-belief task. Asakura and Inui (2016) designed a Bayesian framework that integrates theory-theory and simulation theory for false belief reasoning in the unexpected-contents task. This framework predicted other's belief by the self model and others model which were responsible for simulation-based and theory-based reasoning, respectively. In their opinion, the multiplicative effect of the ability to understand diverse beliefs and knowledge access could predict children's false belief ability. Their model provided good fits to a variety of ToM scale data for preschool children. Rabinowitz et al. (2018) designed a ToM neural network to learn how to model other agents by meta-learning. They constructed an observer who could collect agent's behavioral traces, and its goal was to predict the agent's future behavior. They applied the proposed ToMnet model in simple grid world environments, showing that the observer could model agents effectively and passed Sally Anne Test. And the observer needed not to be able to execute the behaviors itself. O'Laughlin and Thagard (2000) built a connectionist network whose nodes represent the relevant event in the false-belief task, and passed the false-belief task by modifying the connection weight of excitatory links and inhibitory links. Milliez et al. (2014) presented a spatio-temporal reasoning system SPARK, which included a well-designed model of object position hypotheses and generated beliefs. They enabled the robot to passed the Sally-Anne test and performed well in dialog disambiguation. Patacchiola and Cangelosi (2020) proposed a developmental cognitive architecture for trust and ToM in humanoid robots. This architecture was inspired by psychological and biological observations. And it based on an actor-critic (AC) framework, an epigenetic robotic architecture (ERA), and a Bayesian network (BN). These modules represent the functions of the corresponding brain regions in ToM, and they could uncover the detailed mechanisms of trust-based learning in children and robots. Finally, they reproduced psychological experiments with the iCub humanoid robot, and the results are coherent with the real experimental data from children.

### 2.2. False Belief Tasks

There is a wide variety of false-belief tasks, here we introduce two non-verbal unexpected transfer tasks that will be adapted to verify the validity of the model.

Senju et al. (2011) investigated whether 18-month-olds infants would use their own past experience of visual access to attribute perception and consequent beliefs to other people. Infants are divided into two groups, one group wore opaque blindfolds, and another wore trick blindfolds which looked opaque but were actually transparent. The opaque blindfold and trick blindfold looked identical. The test stage is the same as Southgate's as described below. The puppet hid an object in the left box. After the actor wore the same blindfold, the puppet removed the object from the scene. The opaque blindfold group expected the actor to behave according to false belief, and the trick blindfolds did not. Their results show that 18-month-olds used self-experience with the blindfold to assess the actor's visual access and predict their behavior.

Southgate et al. (2007) used an anticipatory looking measure to test whether 2-year-olds infants have the ability of false belief understanding. In the familiarization trials, the puppet hid an object in the left or right box, then left the scene. The actor reached through the corresponding window after doors illuminated with the simultaneous chime. Note that “doors illuminated with simultaneous chime” indicated that the actor was going to reach the object. In one test trial, the puppet hid the object in the left box then move it to the right box. After the actor turned around, the puppet removed the object from the scene. In another test trial, the puppet hid the object in the left box, then the actor turned around. The puppet moved the object to the right box and hid it, then remove the object from the scene. For both test trails, the actor turned back and doors illuminated with simultaneous chime after the object was removed from the scene. Most infants could gaze toward the correct window. Their data demonstrated that 25-month-old infants had the ability of false belief understanding. The details of this experiment were illustrated in the figure of Southgate et al. (2007).

### 2.3. Brain Regions in Theory of Mind

Several brain regions, including the mPFC, bilateral TPJ, and precuneus, have been consistently found to be activated in various mentalizing tasks in healthy individuals (Green et al., 2015). Schurz et al. (2014) meta-analyzed 757 activation foci reported from 73 imaging studies of ToM that involved 1,241 participants, and their meta-analysis contained six different task groups—False belief vs. photo, Trait judgments, Strategic games, Social animations, Mind in the eyes, and rational actions. They found the mPFC and bilateral posterior TPJ were activated in all task groups. In false belief vs. photo stories task group, they found TPJp, IPL, precuneus, posterior cingulate gyrus, mPFC connectivity clusters 3 and 4, ventral parts of the mPFC, anterior cingulate gyrus, right anterior temporal lobe, and adjacent parts of the insula be activated. Molenberghs et al. (2016) conducted a series of activation likelihood estimation (ALE) meta-analyses on 144 datasets (involving 3,150 participants) to address the brain areas that implicated in specific types of ToM tasks. In terms of commonalities, consistent activation was identified in the medial prefrontal cortex and bilateral temporoparietal junction. Schurz and Perner (2015) reviewed nine current neurocognitive theories of how the ToM was implemented in the brain and evaluate them based on the results from a recent meta-analysis by Schurz et al. (2014). From theories about cognitive processes being associated with certain brain areas, they deduced predictions about which areas should be engaged by the different types of ToM tasks. These brain areas contain the mPFC, the pSTS, the TPJ, and the IPL.

## 3. Methods

### 3.1. Architecture of the Brain-ToM Model

**Figure 1:**
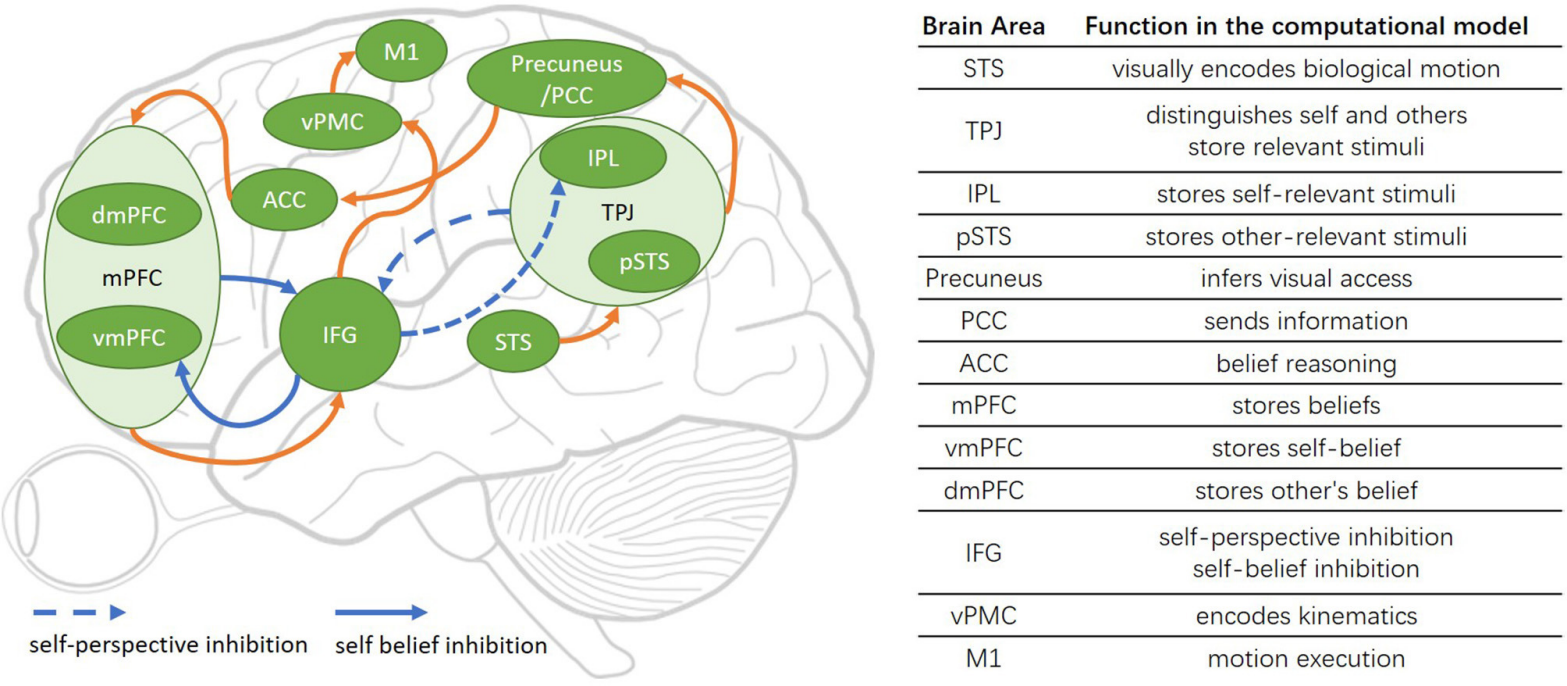
The Brain-ToM model (including major functional brain areas, pathways, and their interactions).

The architecture of the Brain-ToM model is shown in Figure 1.

The STS is sensitive to biological motion, and in our computational model, its function is to visually encode biological motion (Grossman and Blake, 2002).

The TPJ is considered as a crucial area in self-other distinction (Eddy, 2016; Bardi et al., 2017), controls representations relating to the self and other (Eddy, 2016), and involvement in self perspective-taking as well as other perspective-taking (Vogeley et al., 2001; van der Meer et al., 2011). There is no consensus on the anatomical definition of the extent and precise location of the TPJ (Igelstrom and Graziano, 2017), but in general, the TPJ contains two anatomically distinct regions including the IPL and pSTS (Abu-Akel and Shamay-Tsoory, 2011; Schurz et al., 2014; Igelstrom and Graziano, 2017). In our computational model, the TPJ is used to distinguish self and others, store self and other-relevant stimuli, and decide the output sequence of self and other-relevant stimuli.

The IPL is considered as a critical area in distinguishing the self from others and identifying the body ownership in our robot bodily self-model in Zeng et al. (2016, 2017), and some studies have indicated that it will be activated during lower-order self-perception (Schurz and Perner, 2015; Igelstrom and Graziano, 2017). So in our computational model, the IPL is used to store self-relevant stimuli. The pSTS (Frith and Frith, 1999; Schurz and Perner, 2015) is concerned with representing the actions of others and perspective taking (Frith and Frith, 2006). In our computational model, the pSTS is used to store other-relevant stimuli.

The precuneus is often activated during visuo-spatial imagery, episodic memory retrieval, self- processing operations (Cavanna and Trimble, 2006), and retrieving previous experiences (Molenberghs et al., 2016). And a main function of the precuneus in ToM is mental imagery to represent the perspective of another person (Cavanna and Trimble, 2006; Schurz et al., 2013, 2014) or modeling other people's views (Vogeley et al., 2004). In our computational model, the precuneus is the critical area for a machine to learn visual access from its own experience and uses it to infer other people's visual access. The PCC is the caudal part of the cingulate cortex, and the precuneus lies posterior and superior to the PCC (Leech and Sharp, 2014). In our computational model, the PCC receives the information from precuneus and sends it to ACC.

The anterior paracingulate cortex is often considered to be a part of the ACC and is used for representing mental states “decoupled” from reality (Gallagher and Frith, 2003). In our computational model, the ACC is the critical area in acquiring the ability of object permanence and then used it for belief reasoning.

The mPFC contains vmPFC and dmPFC. The vmPFC has typically been associated with self-referential processing, and the dmPFC has typically been associated with others-referential processing (Abu-Akel and Shamay-Tsoory, 2011; Denny et al., 2012; Jiang et al., 2016; Molenberghs et al., 2016). In our computational model, the mPFC is used to store the result of belief reasoning from ACC: the vmPFC stores the result of self-belief reasoning, and the dmPFC stores the other's belief reasoning.

The IFG is a critical area for the inhibition process: self-perspective inhibition and self-belief inhibition. The IFG inhibits self-perspective when self perspective and other-perspective are conflictive (Hartwright et al., 2012, 2015), and is suggested to inhibit self-belief to obtain correct task performance in the false-belief task (Mossad et al., 2016). Another function of IFG is encoding action goals and responding to goal-driven motions (Hamzei et al., 2016). The vPMC encodes kinematics based on motion goals from IFG, the encoded information is sent to M1. M1 encodes the strength and orientation of motion and controls the concrete motion execution (Georgopoulos et al., 1986).

As indicated in Green et al. (2015) and Jiang et al. (2016), the specific roles that brain areas have in the mentalization processes is not clear. Based on the neuroimaging studies as described above, we propose four pathways for robots learning from self-experience and uses it in the false-belief task, they are self-experience learning pathway, motivation understanding pathway, reasoning about one's own belief pathway and reasoning about other people's belief pathway.

The self-experience learning pathways is consist of object permanence learning pathway [Precuneus/PCC → ACC] and the visual access learning pathway [STS → TPJ(IPL) → Precuneus/PCC].

The test pathways are consist of motivation understanding pathway, reasoning about one's own belief pathway, and reasoning about other people's belief pathway.

The motivation understanding pathway is STS → pSTS → TPJ(IPL) → IFG.

The reasoning about one's own belief pathway contains belief reasoning pathway [STS → TPJ(IPL) → Precuneus/PCC → ACC → MPFC(vMPFC)] and the motor response pathway [MPFC(vMPFC) → IFG → vPMC → M1].

The reasoning about other people's belief pathway contains the true belief reasoning pathway and the false belief reasoning pathway.

The true belief reasoning pathway contains belief reasoning pathway [STS → TPJ(pSTS) → Precuneus/PCC → ACC → MPFC(dMPFC)] and the motor response pathway [MPFC(dMPFC) → IFG → vPMC → M1].

The false belief reasoning pathway contains the belief reasoning pathway [STS → TPJ → IFG → TPJ(pSTS) → Precuneus/PCC → ACC → MPFC(dMPFC)] and the motor response pathway [MPFC → IFG → MPFC (dMPFC) → IFG → vPMC → M1].

In the reasoning about other people's belief pathway of false belief reasoning, the conflict between self-and-other perspective in TPJ will activate IFG, then IFG will inhibit self-relevant stimuli in IPL. So the others-relevant stimuli in pSTS will be the first output of TPJ, then it sends to Precuneus/PCC. In the motor response pathway of false belief reasoning, the conflict between self-belief and other's belief in mPFC will activate the IFG, then the IFG inhibits self-belief in vmPFC. So the other's belief in dmPFC will be the output of mPFC, then it will be sent to IFG for encoding action goals.

### 3.2. Concrete Neural Network Architecture

**Figure 2:**
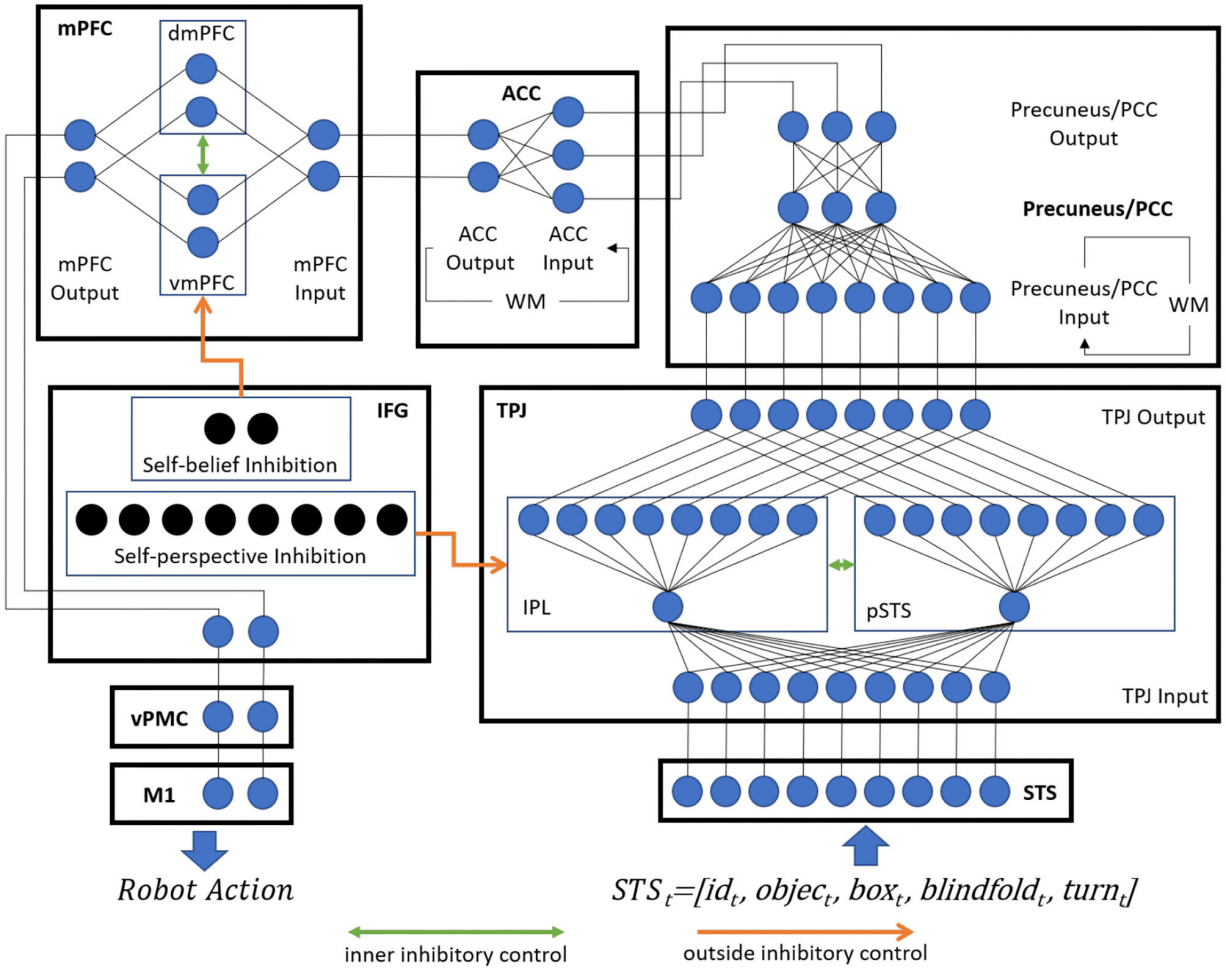
The concrete neural network architecture of Brain-ToM model (WM denotes working memory).

The concrete neural network architecture of the model is shown as Figure 2, and it uses the leaky integrate-and-fire model (LIF) neurons. This section describes (1) the input and output encoding information of different brain areas, (2) the Voltage-driven Plasticity-centric Spiking Neural Networks (VPSNN) used in Precuneus/PCC and ACC for visual access learning and object permanence learning respectively, and (3) the inhibitory control mechanism which was used to select correct output information of TPJ or mPFC.

#### 3.2.1. STS

The STS encodes the processed results of visual perception and body information of the self and others at time *t*.

$$\begin{array}{l} STS_{t}=\left[id_{t},object_{t},box_{t},blindfold_{t},turn_{t}\right] \end{array}$$

We detect this information using traditional template matching methods and represent the result by neurons with an input synaptic current *I* of 1.0 or 0.0. The *id*_*t*_ uses two neurons to represent the identification of self or others. For the identification of *id*_*t*_, we use the Fast R-CNN to recognize others at time *t*. More details could be found in our previous work (Zeng et al., 2017). The *object*_*t*_ and *box*_*t*_ are both tuples consist of object or box identification information and its location information respectively. For the identification of the *object*_*t*_, *box*_*t*_, *blindfold*_*t*_, we first collect their image templates, and then use the traditional template matching method to identify them at time *t*. The location of *object*_*t*_ or *box*_*t*_ is calculated by the distance between the center of the black rectangles and the center of the object or box at time *t*. The *blindfold*_*t*_ uses two neurons to represent the wearing state of the blindfold (wear or not wear) and uses another two neurons to represent whether there is a blindfold at time *t*. Here we define the state of self as wearing a blindfold if the blindfold covers most areas of its visual field, and define the state of others as wearing a blindfold if the blindfold covers the other's face. The *turn*_*t*_ uses two neurons to represent the state of turning. Here the turning-around state of the robot itself is detected by the degree to which its head is twisted, and the turning-around state of the other robot is detected by whether its face or back is recognized.

#### 3.2.2. TPJ

The input information of the TPJ is directly from STS, as

$$\begin{array}{l} TPJ_{input}=STS_{t} \end{array}$$

and the information is divided into self-relevant stimuli and others-relevant stimuli by the *id*_*t*_, and then stored in IPL and pSTS, respectively. The information in IPL, pSTS, and the output of TPJ are encoded as.

$$\begin{array}{l} IPL/pSTS/TPJ_{output}=\left[object_{t},box_{t},blindfold_{t},turn_{t}\right] \end{array}$$

#### 3.2.3. Precuneus/PCC

The Precuneus/PCC is used for visual access learning. Here we use the VPSNN based on our previous work (Tielin et al., 2018) to train the robot to learn visual access.

The input information of Precuneus/PCC contains the current information from the output of TPJ and the previous information from working memory, and it could be represented as

$$\begin{array}{l} Precuneus/PCC_{input_{t}}=TPJ_{output_{t}}+WM_{t} \end{array}$$

where

$$\begin{array}{l} WM_{t}=\zeta \times Precuneus/PCC_{input_{t-1}} \end{array}$$

The ζ is the forgetting factor. In the training stage, the target output of Precuneus/PCC is the perceived location of the object (active the *unseen* signal if no object is detected). The output of Precuneus/PCC is encoded by the input synaptic current *I* of either 1.0 or 0.0 in the perception neurons,

$$\begin{array}{l} Precuneus/PCC_{output_{t}}=\left[location_{1},location_{2},unseen\right] \end{array}$$

There are 160 trials in the training process. Each training trial contains two images collected from the robot as shown in **Figure 4**. The first image is collected when putting the various objects in one location as shown in **Figure 4a**. The second image is collected when the robot is asked “Where is the [object label]?” in three scenes: (1) when the blindfold is interposed (**Figures 4b,c**), (2) when the robot has turned around, (3) when the object is moved to another location. For example, the first image is collected at time *t* − 1, and the second image is collected at time *t*. The input of the VPSNN is *Precuneus*/*PCC*_*inpu*_*t*__*t*__, and the target output of the VPSNN is *Precuneus*/*PCC*_*outpu*_*t*__*t*__. The *Precuneus*/*PCC*_*inpu*_*t*__*t*__ receives two inputs: one is the raw *TPJ*_*outpu*_*t*__*t*__ and the other is the *Precuneus*/*PCC*_*inpu*_*t*__*t*−1__ with a forgetting factor. In each training trial, there is no previous information from working memory when collecting the first image, so the *Precuneus*/*PCC*_*inpu*_*t*__*t*−1__ is equal to the *TPJ*_*outpu*_*t*__*t*−1__. The *Precuneus*/*PCC*_*outpu*_*t*__*t*__ is the perceived location of the object at time *t*. The robot trains the self-experience of visual access to the wearing of the blindfold or the turning around of the robot in the process of visual access learning, then uses it to infer itself and other robot's visual access in the Opaque-and-Transparent Blindfold Test and Turn Around Test.

#### 3.2.4. ACC

The ACC is used for object permanence learning. Here we use the VPSNN to train the robot to learn object permanence. The input information of ACC contains the current information from the output of Precuneus/PCC and the previous information from working memory, and it could be represented as

$$\begin{array}{l} ACC_{input_{t}}=Precuneus/PCC_{output_{t}}+WM_{t} \end{array}$$

where

$$\begin{array}{l} WM_{t}=\zeta \times ACC_{output_{t-1}} \end{array}$$

The ζ is the forgetting factor. In the training stage, the target output of ACC is the location of the object. The output of ACC is encoded by the input synaptic current *I* of either 1.0 or 0.0 in the related neurons, i.e.,

$$\begin{array}{l} ACC_{output_{t+1}}=\left[belief_{location_{1}},belief_{location_{2}}\right] \end{array}$$

**Figure 3:**
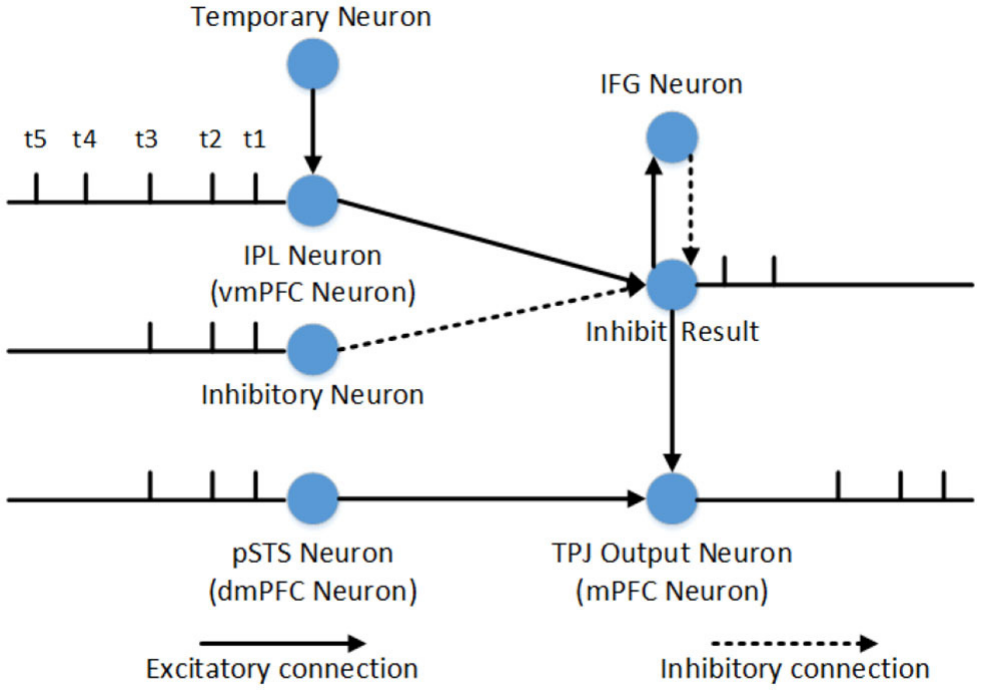
Inhibitory control of one single neuron in reasoning about other's belief. At time t1, t2, and t3, the firing pattern of pSTS and IPL are identical, which means that the other-relevant stimuli and self-relevant stimuli are identical. The inhibitory neurons can inhibit self-relevant stimuli successfully. But at time t4 and t5, the other-relevant stimuli and self-relevant stimuli are in conflict with each other, and the inhibitory neurons cannot inhibit self-relevant stimuli. The inhibit result neurons will fire and stimulate IFG activation, while IFG activation will inhibit the firing of inhibit result neuron. The inhibit result neurons will combine with neurons in pSTS to generate other-relevant stimuli output firstly. Then the temporary neuron stimulates IPL, because the electrovital currents in pSTS and the inhibitory neuron is zero at this moment, self-relevant stimuli will be the second output of TPJ. Compared to the process of self-perspective inhibition, the only difference is that the inhibitory control in TPJ is used to decide the sequence of information output, and the inhibitory control in mPFC is used to decide which belief to export. Therefore, the process of self-belief inhibition does not require the involvement of temporary neuron.

There are 50 trials in the training process. Each training trial contains three images collected from the visual sensor, when (1) the various objects are put in one location (**Figure 5b**), (2) an object is hidden in the box (**Figure 5c**), and (3) the box is moved away (**Figure 5d**). For example, the first, second, third image is collected at time *t* − 1, *t*, *t* + 1, respectively. The input of the VPSNN is *ACC*_*inpu*_*t*__*t*__, and the target output of the VPSNN is *ACC*_*outpu*_*t*__*t*+1__. The *ACC*_*inpu*_*t*__*t*__ receives two inputs: one is the raw *Precuneus*/*PCC*_*outpu*_*t*__*t*__ and the other is the *ACC*_*outpu*_*t*__*t*−1__ with a forgetting factor. The *ACC*_*outpu*_*t*__*t*−1__ is the perceived location of the object at time *t* − 1. To train the ability of object permanence in the robot, we make the robot always perceive the location of the object at the end of each training trial. The *ACC*_*outpu*_*t*__*t*+1__ is the perceived location of the object at time *t* + 1. The robot trains itself a belief that objects are still where it has last located them, even they are out of its field of visual perception, and then uses it to infer itself and other robot's belief in the Opaque-and-Transparent Blindfold Test and Turn Around Test.

We train the visual access learning in Precuneus/PCC first, then the object permanence learning in ACC.

#### 3.2.5. mPFC

The input of mPFC is identical to the output of ACC and distinguishes between the self-belief and other-belief by the source of the information: IPL or pSTS. The vmPFC and dmPFC both use two neurons to store the self-belief and other-belief about the location of the object.

$$\begin{array}{l} \begin{array}{ccc} & mPFC_{input_{t}}=ACC_{output_{t}} & \\ & dmPFC_{t}=mPFC_{input_{t}}\;if\;the\;source\;is\;IPL\\ & vmPFC_{t}=mPFC_{input_{t}}\;if\;the\;source\;is\;pSTS \end{array} \end{array}$$

The output of mPFC depends on the questions, which are set as follows: if the question is “Where is the ladybird according to the blue robot?” the other-belief stored in dmPFC tries to be the output of mPFC; if the question is “Where is the ladybird according to yourself?” the self-belief stored in vmPFC tries to be the output of mPFC.

$$\begin{array}{l} mPFC_{output_{t}}=\left\{ \begin{array}{ccc} dmPFC_{t}\; & if\;the\;question\;is\;“Where\;is\;the\; & \\ & ladybird\;according\;to\;the\;blue\;robot”\\ vmPFC_{t}\; & if\;the\;question\;is\;“Where\;is\;the\\ & ladybird\;according\;to\;yourself?” \end{array}\right. \end{array}$$

#### 3.2.6. IFG

In the proposed model, IFG receives inputs from three sources: (1) the inhibit result neurons in TPJ that could stimulate IFG for self-perception inhibition, (2) the inhibit result neurons in mPFC that could stimulate IFG for self-belief inhibition, and (3) some other neurons in mPFC that could stimulate IFG to encode the action goal.

$$\begin{array}{l} IFG_{input_{t}}=\left\{ \begin{array}{cc} I_{inhibit\;result\;neuron\;TPJ} & self-perception\;inhibition\\ I_{inhibit\;result\;neuron\;mPFC} & self-belief\;inhibition\\ mPFC & encode\;the\;action\;goal \end{array}\right. \end{array}$$

IFG uses the same number of neurons as IPL and as vmPFC to inhibit self-perception information and self-belief information, respectively, and uses another two neurons to encode the action goal, which is later sent to vPMC to control the robot's actions. The details of the inhibitory control mechanism of IFG could be found in section 3.4.

With the exception in the above mentioned evaluation, the synaptic plasticity only takes place in Precuneus/PCC and ACC in the process of training, while the weights of the other connections between various areas remain unchanged in the experiment.

### 3.3. VPSNN

For the mathematically modeling of brain regions such as Precuneus/PCC and ACC, here we select a standard VPSNN model (Tielin et al., 2018), which is a shallow feed-forward SNN and may well simulate input-output signals with the integration of supervised learning (with an additional teaching signal given directly to the output layer neurons) and unsupervised learning (tuned with biologically plasticity principles, e.g., STDP, and homeostatic membrane potential).

Two three-layer SNN architectures are designed for Precuneus/PCC (with 24 input neurons, three hidden neurons, and two output neurons) and ACC (with three input neurons, three hidden neurons, and two output neurons), respectively, as shown in Figure 2. The VPSNN includes four steps, namely: feed-forward information (including both membrane potential and spikes) propagation, unsupervised homeostatic state learning, supervised last layer learning, and passively updating synaptic weights based on STDP rules. In this paper, we take advantage of these four steps for the fast network tuning and update the methodologies of giving teaching signals from single SNN to two SNNs together for the better model integration.

#### 3.3.1. The LIF Neuron Model

(1) $$\begin{array}{r} \tau_{m}\frac{dV}{dt}=-\left(V-V_{L}\right)-\frac{g_{E}}{g_{L}}\left(V-V_{E}\right) \end{array}$$

(2) $$\begin{array}{r} \tau_{E}\frac{dg_{E}}{dt}=-g_{E}+\eta \underset{j\in N_{E}}{\sum }w_{j,i}\delta_{t} \end{array}$$

The basic neuron model in VPSNN is the LIF model, which describes the dynamics of the membrane potential of *V* and synaptic-weight-related *g*_*E*_, as shown in Equations (1) and (2). Once the pre-synaptic neurons fire, there is a non-linear increment of *g*_*E*_, which will then propagate into *V*. The *g*_*L*_ is leaky conductance, *V*_*L*_ is leaky potential, τ_*m*_ and τ_*E*_ are conductance decay, η is the learning rate, and *V*_*E*_ is reversal potential.

#### 3.3.2. The Feed Forward Propagation

The information propagation in the LIF neuron is slower compared with giving input directly into *V*. However, this is a specially designed procedure that will make the network-tuning focusing more on the homeostatic membrane potential adjustment and STDP learning. The information (especially the membrane potential) will be propagated from pre-synaptic neurons (e.g., *V*_*j*_) into the post-synaptic neurons (e.g., *V*_*i*_), and the whole feed-forward procedure $V_{i}^{FF}$ is shown in Equation (3), in which the *V*_*th*_ is the firing threshold of the neurons.

(3) $$\{\begin{array}{l} \tau_{m}\frac{dV_{i}}{dt}=-\left(V_{i}-V_{L}\right)-\frac{g_{E}}{g_{L}}\left(V_{i}-V_{E}\right)\\ \tau_{E}\frac{dg_{E}}{dt}=-g_{E}+\eta \sum_{j}^{N}w_{j,i}V_{j}\\ V_{i}=V_{L},\;T_{ref}=T_{0}\;if\left(V_{i}>V_{th}\right)\\ V_{i}^{FF}=V_{i} \end{array}\;$$

#### 3.3.3. Unsupervised Homeostatic Membrane Potential Learning

The basic homeostatic mechanism occurs in the input-output balance of the single neuron, described as the Equation (4).

(4) $$\begin{array}{r} \Delta E_{i}=V_{i}-\left(\overset{N}{\underset{j}{\sum }}w_{j,i}V_{j}-V_{th,i}\right) \end{array}$$

The entire network homeostatic state can be represented as the addition of all of the neurons in each layer, i.e., $\Delta E=\underset{i\in N}{\sum }\Delta E_{i}$. Moreover, after the calculation (the detailed methodologies are shown in paper Tielin et al., 2018), the homeostatic membrane potential $V_{i}^{Homeo}$ can be updated according to Equation (5). The η_*i*_ is the learning rate.

(5) $$\begin{array}{r} \Delta V_{i}^{Homeo}=-\eta_{i}\frac{V_{i}-\left(\overset{N}{\underset{j}{\sum }}w_{j,i}V_{j}-\overset{N}{\underset{j}{\sum }}V_{th,i}\right)}{-\left(V_{i}-V_{L}\right)-\frac{g_{E}}{g_{L}}\left(V_{i}-V_{E}\right)} \end{array}$$

With the integration of Equations (3) and (5), the update rule of each neuron state *V*_*i*_ is shown in Equation (6). The *t* is the training time slot and *T* is the total training time in SNN learning.

(6) $$\begin{array}{r} \Delta V_{i}=\frac{t}{T}\Delta V_{i}^{FF}+\left(1-\frac{t}{T}\right)\Delta V_{i}^{Homeo} \end{array}$$

#### 3.3.4. Supervised Last Layer Learning and STDP-Based Weights Consolidation

An additional teaching signal will be given to the network for guiding the proper network output. Here we add teaching signals into the last layer of SNN in the training procedure, as shown in Equation (7), in which *V*_*T*_ is teacher signal state, η^*c*^ is the learning rate.

(7) $$\begin{array}{r} dV_{i}=-\eta^{c}\left(V_{i}-V_{T}\right) \end{array}$$

STDP rules (Bi and Poo, 2001; Dan and Poo, 2004; Bengio et al., 2015a,b) are further used for the knowledge consolidation from the membrane potential to synaptic weights, e.g., the synaptic weights could be passively updated by the changes of the pre- and post-synaptic neuron states. The function is as shown in Equation (8), in which the $V_{i}^{{}^{\prime }}$ is the derivative value of *V*_*i*_.

(8) $$\begin{array}{r} \Delta w_{j,i}\propto V_{j}V_{i}^{{}^{\prime }} \end{array}$$

### 3.4. Inhibitory Control Mechanism

The inhibitory control is used to select correct output information of TPJ or mPFC, and it can be divide into inner and outside inhibitory control. The inner inhibitory control cannot inhibit predominant information from self when the related information of self and others is conflictive, so we use the outside inhibitory control from IFG to inhibit the predominant information. Inhibitory control of one single neuron is shown in Figure 3. The IPL neurons (or vmPFC neurons) and pSTS neurons (or dmPFC neurons) receive electrovital currents of self-relevant stimuli and other-relevant stimuli, respectively. The input of inhibit neurons and temporary neurons depend on reasoning about other's belief (contains self-perspective inhibition and self-belief inhibition) or self-belief (contains other-perspective inhibition and other-belief inhibition), and the former is used to inhibit stimuli, the later is used to temporarily store uninhibited stimuli. In the process of self-perspective inhibition or self-belief inhibition, the input electrovital currents of inhibit neuron are equal to other-relevant stimuli, and the temporary neuron is equal to self-relevant stimuli (only in TPJ). So other-relevant stimuli will be the first output, and self-relevant stimuli will be the second output in TPJ, and other's belief will be exported in mPFC. In the process of other-perspective inhibition and other-belief inhibition, the inhibit electrovital currents are very big that it can completely inhibit other-relevant stimuli, and the temporary neuron is equal to other-relevant stimuli (only in TPJ). Therefore, in TPJ, self-related stimulus will be output first, other-related stimulus will be output second, and then self-belief will be output in mPFC.

## 4. Experiments

In this section, we introduce the experimental settings and the result of our proposed model.

### 4.1. Experimental Settings

We deploy the computational model to humanoid robotics and use the Opaque-and-Transparent Blindfold Test and Turn Around Test to validate the Brain-ToM model. Furthermore, we test the effect of self-experience, maturation of correlate brain areas (e.g., calculation capability) and their connections (e.g., inhibitory control) on the performance of participant robot in Opaque-and-Transparent Blindfold Test.

#### 4.1.1. Opaque-and-Transparent Blindfold Test

The Opaque-and-Transparent Blindfold Test is adapted from Senju et al. (2011).

**Figure 4:**
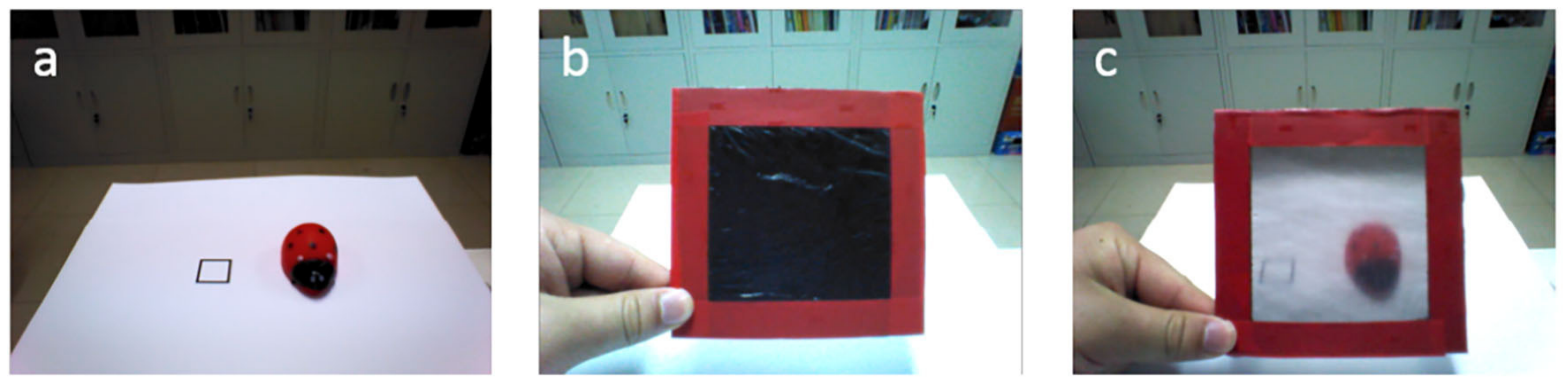
Visual access learning of blindfold from self-experience. **(a)** An object is put on either of the black rectangles. **(b)** Visual inputs of the opaque blindfold group (with Movie S1). We interpose blindfold between the eyes of the robot and the object, then ask it “Where is the [object label]?” The robot will reply with the location of the object or with the fact that it did not see it. **(c)** Visual inputs of the transparent blindfold group (with Movie S2). The process is the same as the opaque blindfold group.

We enable all the robots to learn the visual access of blindfold from self experiences, as the infants' experience in Senju's experiment. In the proposed model, this process takes place in Precuneus (Vogeley et al., 2004; Cavanna and Trimble, 2006; Schurz et al., 2013, 2014). The robots are divided into two groups—the opaque blindfold group and the transparent blindfold group. The opaque blindfold and transparent blindfold look identical, at least the robot cannot distinguish them from appearance, but the transparent blindfold can make the robot who wears it see through, and the opaque blindfold cannot. Figure 4 presents the visual inputs of the robot in the opaque blindfold group (with Movie S1) and the transparent blindfold group (with Movie S2). In this stage, the robots could not observe other robots wearing the blindfold, as the infants in Senju's experiment, so they have no opportunity to learn the property of the blindfold from the third-person point of view.

**Figure 5:**
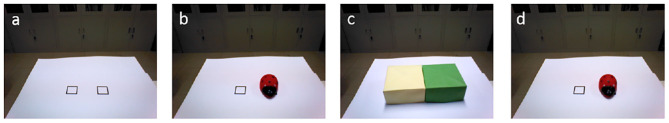
Object permanence learning from self-experience. **(a)** The black rectangles are used to indicate the candidate positions of the object. **(b)** An object is put on either of the black rectangles, the robot can detect the location of the object—left side or right side. **(c)** The yellow box and the green box are used to hide the object. The robot cannot perceive the object in its visual field, hence cannot find it. It is similar to the early stages in Piaget's Stages of Object Permanence. **(d)** The boxes are removed, and the robot can perceive the object's location in its visual field.

We suggest that the ability of learning object permanence is the prerequisite for the ToM. As indicated in Piaget and Cook (1952) and Bruce and Muhammad (2009), Piaget defined six developmental stages of object permanence. During the early stages (Stage I, Stage II, Stage III), children failed to find a hidden object. During Stage IV (8–12 months) children can retrieve an object when its concealment is observed. But they cannot find the object when it is continuously moving. During Stage V (12–18 months), the children can retrieve an object when it is hidden several times within his or her view. In summary, when an object was hidden in location A and then hidden in location B, the children would try to find the object in location A during Stage IV and would try to find it in location B during Stage V. With similar principles, here we enable all of the robots in the experiment acquire the ability of learning object permanence from their own self experiences, and in our model, ACC acts as a central role to realize this cognitive function (Gallagher and Frith, 2003). Figure 5 shows the visual inputs of the robots in this process.

**Figure 6:**
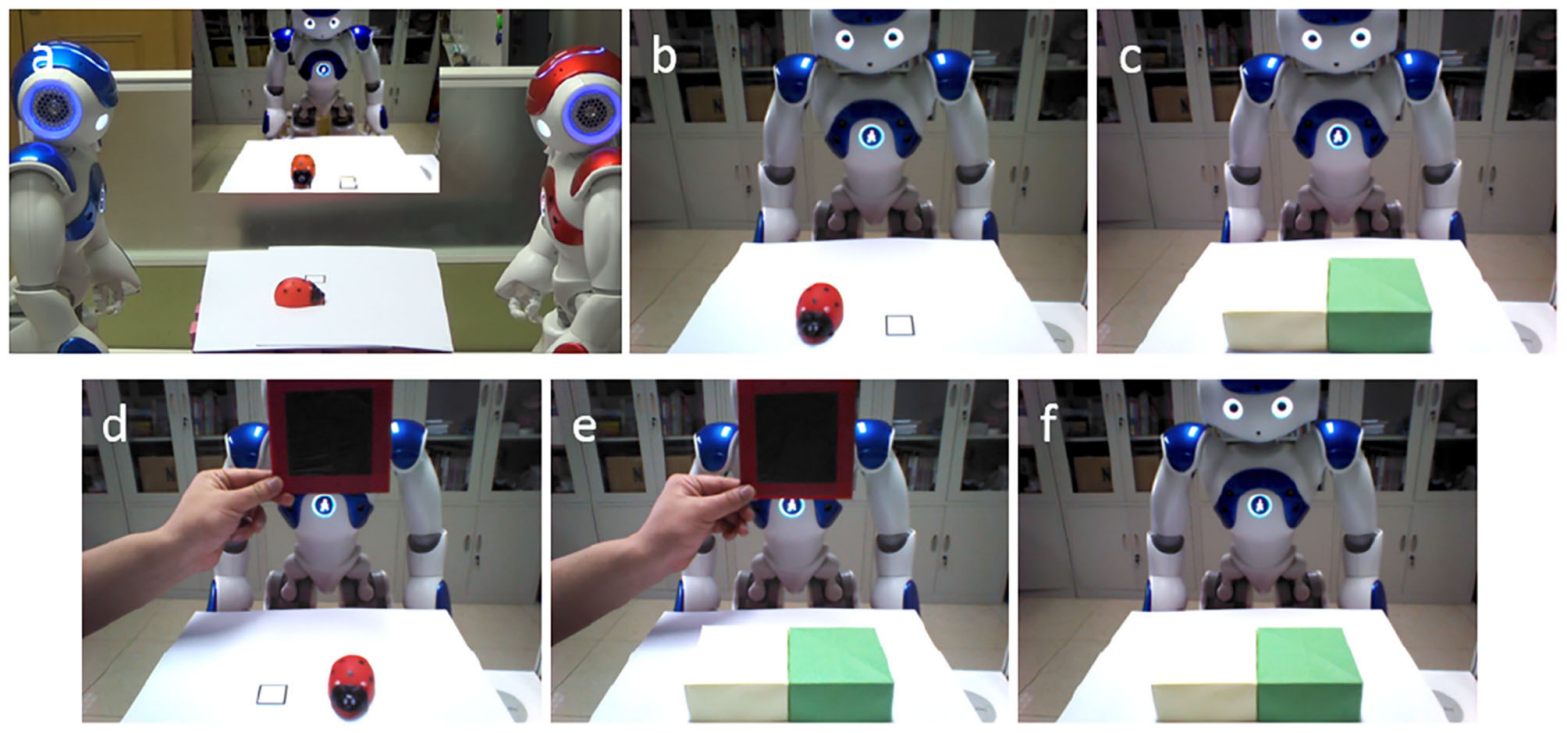
Visual inputs of participant robot in the test stage. **(a)** The blue robot in the left is the actor, and the red robot in right is the participant who should infer the actor's belief. The middle screen in the **(a)**, and the remaining figures are the visual inputs of the participant robot. **(b)** An object (ladybird) is put on the left black rectangle. **(c)** The ladybird is hidden in the yellow box. **(d)** The blindfold is interposed between the actor (the blue robot) and the object (ladybird), and the object is moved to the right side. **(e)** The ladybird is hidden in the green box. **(f)** Finally, the blindfold is removed.

In the test stage, participant robots use the Brain-inspired Robot Bodily Self Model which we proposed in Zeng et al. (2016, 2017) to distinguish self and others. As in the experiment of Senju et al. (2011), the actor robot will try to find the hidden object in the box before the final test. By this way, the participant robot could understand that the actor has the same cognitive ability (e.g., visual ability) and the goal of the actor robot (Movie S3). In the final test, the opaque blindfold group and the transparent blindfold group are tested with the same process as shown in Figure 6. Then the participant robot be asked two questions: “Where is the ladybird according to the blue robot?” and “Where is the ladybird according to yourself?” We determine whether the robot can pass the task by detecting the direction of the finger which makes the results more intuitive.

#### 4.1.2. Turn Around Test

The Turn Around Test is adapted from Southgate et al. (2007).

**Figure 7:**
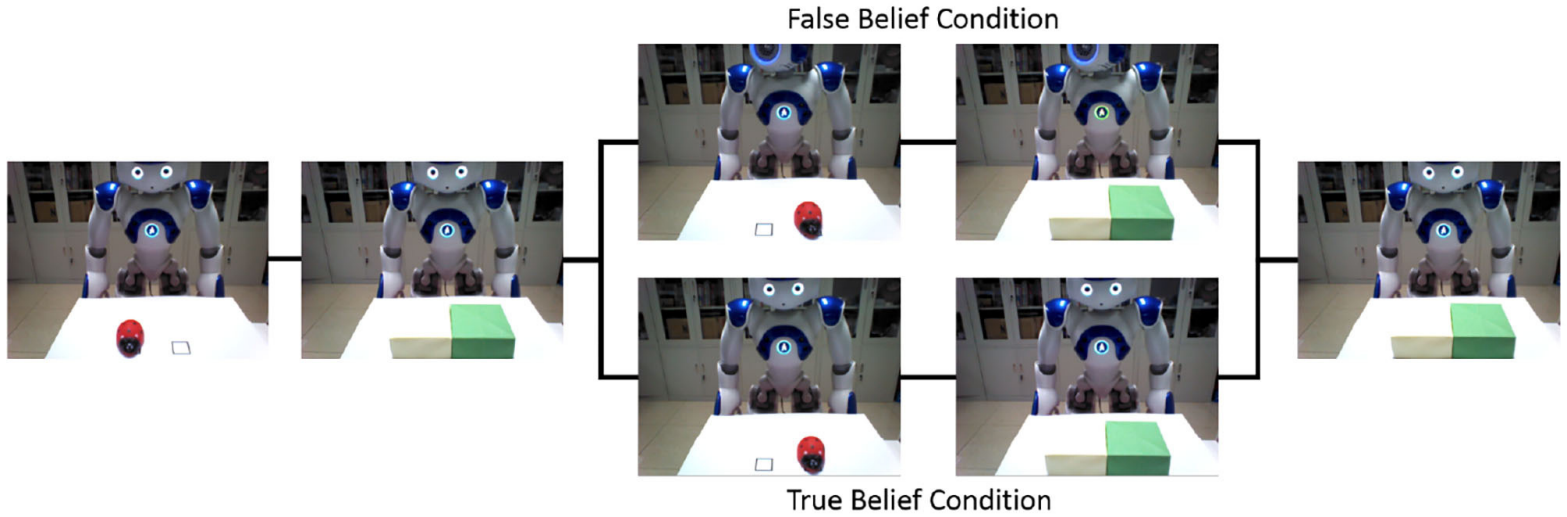
Turn Around Test. An object (ladybird) is put on the left black rectangle and hidden it in the yellow box firstly. In the false belief condition, the object was moved to the other box when the actor robot turned around (Movie S6). And in true belief task, the actor robot did not turn around when the object was moved to the other box (Movie S7).

The robot learns the visual access of turning around from self-experience. The Turn Around Test is similar to the Opaque-and-Transparent Blindfold Test. The diversity of belief is caused by different blindfolds in the Opaque-and-Transparent Blindfold Test, and in Turn Around Test, it is caused by the behavior of turn around. The visual inputs of the participant robot are shown in Figure 7. As with the Opaque-and-Transparent Blindfold Test, the participant robot also be asked two questions: “Where is the ladybird according to the blue robot?” and “Where is the ladybird according to yourself?” And we determine whether the robot can pass the task by detecting the direction of the finger.

#### 4.1.3. Maturation Test

The ability for the ToM comes with individual development process (Grosse Wiesmann et al., 2017). Grosse Wiesmann et al. (2017) discussed the influence of white matter structure on ToM by tract-based spatial statistics analysis and probabilistic tractography. They found that “the developmental breakthrough in false belief understanding is associated with age-related changes in local white matter structure in temporoparietal regions, the precuneus, and medial prefrontal cortex, and with increased dorsal white matter connectivity between temporoparietal and inferior frontal regions.” And they thought “the emergence of mental state representation is related to the maturation of core belief processing regions and their connection to the prefrontal cortex” (Grosse Wiesmann et al., 2017). But their research focused on the 3- and 4-year-old children in the explicit false-belief tasks, and did not include younger infants who cannot pass the implicit false-belief task. They did not test whether this finding is also associated with an implicit task, because of the difficulties in performing MRI with toddlers.

Although the developmental neural basis for the implicit false-belief task is still not very clear, we hypothesize that the developmental process in implicit false belief understanding is relevant with explicit one, and will also be associated with the maturation of correlate brain areas and their connections. We aim to test this hypothesis by our computation model, and apply it to the Brain-ToM model that we developed for machine intelligence.

The maturation of correlate brain areas could be regarded as calculation capability in our model, and the calculation capability increases with the maturation of brain areas. The calculation capability in this model is proportional to the number of neurons in the hidden layer. We simulate immature Precuneus/PCC by reducing the number of the neurons in its own hidden layer, then verify the effect of this condition on the performance of the participant robot.

The maturation of the connection between brain areas is critical for information transmission and information integration, especially inhibitory connection and control. The inhibitory control is generally considered as a key mechanism in false-belief task (Leslie and Polizzi, 1998; Scott and Baillargeon, 2017), and we think that the maturation of connections between IFG and TPJ, IFG and vmPFC are the neural basis of self-perspective inhibition and self-belief inhibition, respectively. We simulate immature connections between IFG and TPJ, IFG and vmPFC by set the synaptic weights as 0, then verify the effect of this condition on the performance of the participant robot.

### 4.2. Experimental Results and Analyses

In this section, we present the results of our model. Besides, we analyze the temporal and spatial activation of different brain areas during different tasks, the effect of self-experience, maturation of correlate brain areas (e.g., calculation capability) and their connections (e.g., inhibitory control) on the performance of participant robot in Opaque-and-Transparent Blindfold Test.

#### 4.2.1. Opaque-and-Transparent Blindfold Test

The opaque blindfold group can be regarded as the false belief condition, as the actor robot's belief is inconsistent with the representations of reality. When asking the participant robot “Where is the ladybird according to the blue robot?” the participant robot will point to the yellow box on the left side. And when asking the participant robot “Where is the ladybird according to yourself?” the participant robot will point to the green box on the right side (Movie S4).

The transparent blindfold group can be regarded as the true belief condition, as the actor robot's belief is consistent with the representations of reality. When asking the participant robot the upper two questions, for both of them, the participant robot will point to the green box on the right side (Movie S5).

We repeat this experiment 20 times, the robot could pass the task every time, and we calculate the mean value and standard deviation of time consumption in different brain areas. To make the results more visible and clear, the data from STS, Precuneus/PCC, ACC, vPMC/M1 are excluded, because their time consumption is similar in different tasks. We select the time consumption in TPJ, IFG, and mPFC as examples. The time consumption of false belief reasoning (87.7 ms) is longer than true belief reasoning (69.8 ms). And the time consumption of reasoning about other's belief (78.8 ms) is longer than self-belief (15.2 ms). In the process of reasoning about self-belief, the time consumption of false belief condition (15.4 ms) and true belief condition (15 ms) are similar. In traditional true belief task, the time consumption will be shorter. The perception in TPJ is identical, hence the IFG will not be activated in this task and the time consumption is reduced.

**Figure 8:**
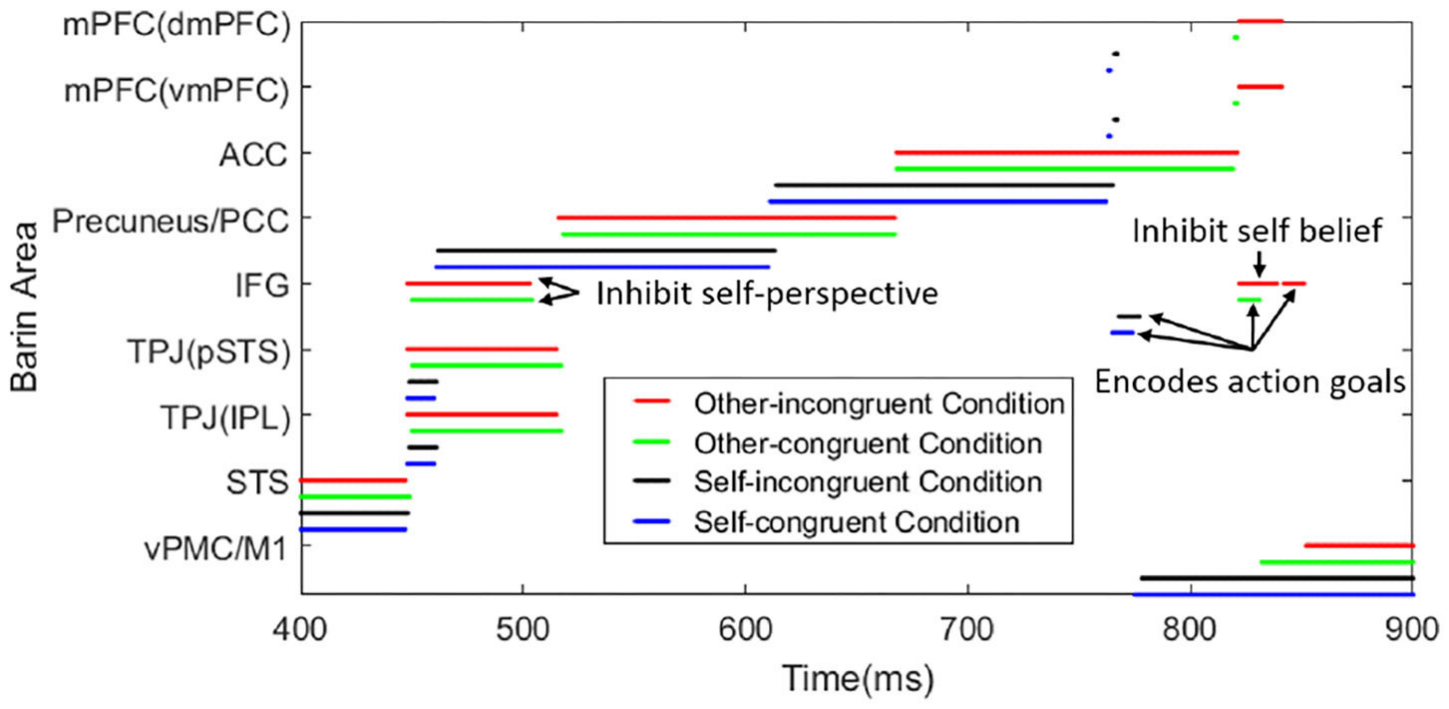
The temporal and spatial activation of different brain areas during different tasks. The time consumption in STS (visually encoding biological motion), Precuneus/PCC (inferring visual access), ACC (belief reasoning), IFG (encoding action goals), and vPMC/M1 (encoding kinematics/motion execution) are similar in different tasks. In the process of TPJ activation (deciding the output sequence of self and other-relevant stimuli) in other-incongruent condition (red line) and other-congruent condition (green line), IFG is activated to inhibit self-perspective. In the process of mPFC activation (deciding belief for motor response) in other-incongruent condition, IFG is activated to inhibit self-belief. In the tasks of self-incongruent condition (black line) and self-congruent condition (blue line), the IFG is only activated in the process of encoding action goals. The value of total time consumption is self-congruent condition < self-incongruent condition < other-congruent condition < other-incongruent condition. To show the function of IFG easily, we make the arrow mark in the figure.

Here we provide the time consumption in different tasks. Based on the belief about the object location, the task can be divided into reasoning about actor robot's false-belief task (other-incongruent condition) and true belief task (other-congruent condition), reasoning about participant robot's own belief task which contains self-incongruent condition (self-belief is divergent from other's) and self-congruent condition (self-belief is corresponding with other's). Figure 8 shows the temporal and spatial activation of different brain areas during different tasks. This process only contains the perception conflict stage (as shown in Figure 6d) and motion response stage which have critical differences in different tasks. The time consumptions in STS and vPMC of different tasks are similar, about 448 and 500 ms, respectively. So we select 400-900 ms to show the process and the difference in different tasks. Reasoning about other's belief in the transparent group can be regarded as true belief task, but it must be noted that, the information from self-perspective and other-perspective are identical in tradition true belief task, while they conflict with each other in this task which is originally from Senju et al. (2011) based on human studies of 18-month-olds. For example, in the transparent blindfold task, the participant robot perceived from self-perspective that “I (participant robot) saw the object moved to the green box without blindfold” and the participant robot perceived from other-perspective that “the actor robot saw the object moved to the green box with blindfold,” so the information is conflictive in this task. And in traditional true belief task, the information is identified as “I (participant robot) saw the object was moved to the green box” and “the actor saw the object was moved to the green box” without the difference caused by the transparent blindfold.

Our focus is the activation sequence of brain areas and reaction time in the various task, such as the other-incongruent condition spends more time than self-congruent condition rather than the numerical value of time consumption, these result is consistent with the functional neuroimaging studies in Mossad et al. (2016) and Dohnel et al. (2012).

In the process of inferring visual access which corresponds to Figure 6d, even though the visual inputs in both groups are identical, the output is different when the participant robot infer other's visual access with different self-experience. In other words, when inferring visual access of another person by self-experience, the opaque blindfold group will know the actor cannot see the moving object, and the transparent blindfold group will know the actor can. When inferring self visual access, the perception of visual inputs and the result of precuneus are identical in both groups.

In the process of inferring other's visual access, the IFG will not be activated when self-perspective and other-perspective is identical, as shown in Figures 6b,c,f. If the self-perspective and other-perspective are in conflict with each other in the process of reasoning about other's belief, the IFG will be activated to inhibit the information of self-perspective, as shown in Figures 6d,e.

In the process of motor response in reasoning about other's belief, the IFG will inhibit self-belief if the beliefs are conflictive. So the other's belief in dmPFC will be the output of mPFC. Then IFG receives input from mPFC and encodes action goals to control vPMC to action.

In addition, we also test the effect of blindfold position in this task. In the visual access of blindfold learning stage, we add a new phase: we put the blindfold on the desk or interpose it at others position to make that both the blindfold and object can be perceived by the participant robot, and we also ask the question “Where is the [object label]?” In the test stage, we put the blindfold on the desk or interpose it at other positions where the actor's visual inputs are not blocked. Both of the groups can infer the visual access of actor robot correctly, and conclude that the actor robot could see the object move to the right side. And self-belief is corresponding to other's belief in both groups. This additional test could prove that the actor robot does not use low-level features such as whether the blindfold exists when inferring other's mental state, and it also proves the effect of the bodily model in this task.

#### 4.2.2. Turn Around Test

In the false belief condition, the actor robot's belief is inconsistent with the representations of reality. When asking the participant robot “Where is the ladybird according to the blue robot?” the participant robot will point to the yellow box on the left side. And when asking the participant robot “Where is the ladybird according to yourself?” the participant robot will point to the green box on the right side (Movie S6).

In the true belief condition, the actor robot's belief is consistent with the representations of reality. When asking the participant robot the upper two questions, for both of them, the participant robot will point to the green box on the right side (Movie S7).

We repeat this experiment 20 times, the robot could pass the task every time. The mechanism of Turn Around Test is similar to the Opaque-and-Transparent Blindfold Test, the only difference is that the self-perspective and other-perspective are identical in true belief task of Turn Around Test. So IFG was not activated in this stage. We select the time consumption in TPJ, IFG, and mPFC as examples. The time consumption of false belief reasoning (87.3 ms) is longer than true belief reasoning (16.1 ms). And the time consumption of reasoning about other's belief (51.7 ms) is longer than self-belief (15.3 ms). In the process of reasoning about self-belief, the time consumption of false belief condition (15.4 ms) and true belief condition (15.2 ms) are similar.

#### 4.2.3. Maturation Test

The maturation of correlate brain areas will be regarded as calculation capability in our model, and the calculation capability increases with the maturation of brain areas. The calculation capability in this model is proportional to the number of neurons in the hidden layer. For example, If the neuron number involved in the calculation of the hidden layer in Precuneus/PCC is two or fewer, even though participant robot can learn visual access of blindfold, it failed in inferring other's visual access. As indicated in Myowa-Yamakoshi et al. (2011), 8-month-old infants and 12-month-old infants had experienced being blindfolded, when they saw a blindfolded actor did a successful goal-directed action which normally could not succeed with a blindfold, 12-month-old infants will look longer, but 8-month-old infants will not. We think that with the maturation of correlate brain areas as well as their connections, more neurons and synaptic connections will be included in the task processing.

**Figure 9:**
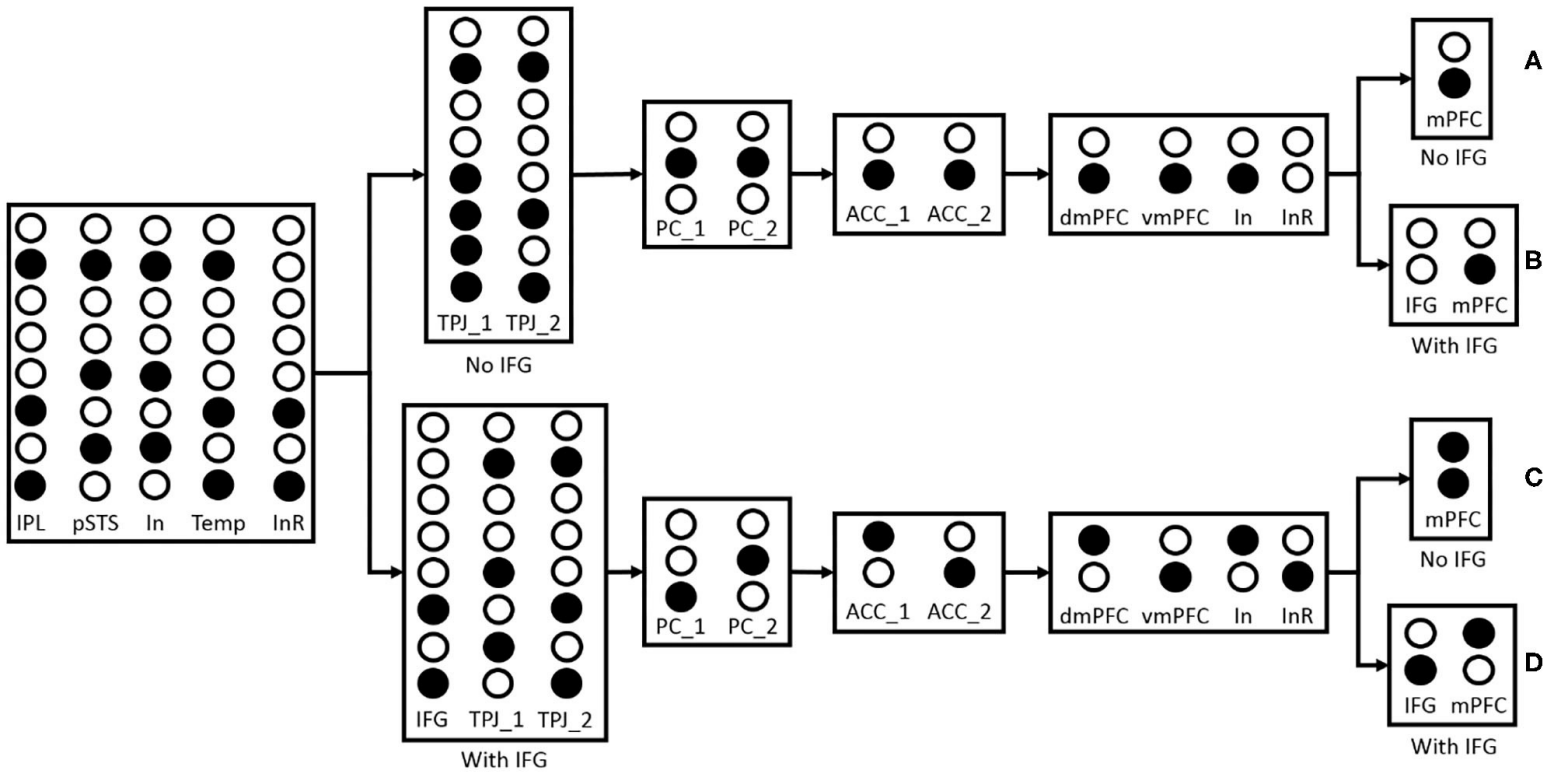
The effect of IFG connection in the false-belief task. The solid circles indicate that the neurons have been activated. “No IFG” means the connection between IFG and TPJ or IFG and vmPFC is immature, and ‘With IFG' means the connection between IFG and TPJ or IFG and vmPFC is mature. **(A)** Both of the connections between IFG and TPJ, IFG and vmPFC are immature. The first output of TPJ (TPJ_1) is different from IPL or pSTS because the self-perspective inhibition failed which caused by the immature connection between IFG and TPJ, and the second output (TPJ_2) is identical with IPL. The inferring visual access result of PC_1 and PC_2 in Precuneus/PCC are identical, and the belief reasoning result of ACC_1 and ACC_2 in ACC are identical. In the process of motor response, the InR neurons have not been activated because the belief in dmPFC and vmPFC are identical, so the output of mPFC is identical with dmPFC. The behavior of the participant robot to predict the actor robot's action is the same as the action caused by self-belief, so it failed in the false-belief task. **(B)** The connection between IFG and TPJ is immature, and the connection between IFG and vmPFC is mature. The participant robot also failed in this task because the result of inferring other's visual access is wrong which causes by the immature connection between IFG and TPJ. **(C)** The connection between IFG and TPJ is mature, and the connection between IFG and vmPFC is immature. The activated InR neurons activate IFG to inhibit self-perspective. The TPJ_1 and TPJ_2 could be output correctly. The participant robot could infer other's belief correctly, but it cannot inhibit the effect of self-belief without IFG. Both of the two candidate responses are activated when asked the question “Where is the ladybird according to the blue robot.” The participant robot will tend to select action directed by self-belief and failed in the false-belief task. **(D)** Both of the connections between IFG and TPJ, IFG, and vmPFC are mature. With the connection to IFG, the participant robot will inhibit self-perspective in TPJ and inhibit self-belief in mPFC, then succeed in this task.

The maturation of the connection between brain areas is critical for information transmission and information integration, especially inhibitory connection and control. The inhibitory control is generally considered as a key mechanism in false-belief task (Leslie and Polizzi, 1998; Scott and Baillargeon, 2017), and we think that the maturation of connections between IFG and TPJ, IFG and vmPFC are the neural basis of self-perspective inhibition and self-belief inhibition respectively. As shown in Figure 9, the inhibitory control uses inhibitory neurons and temporary neurons which store information temporarily for the selection of correct output information of TPJ or mPFC. In the process of reasoning about other's belief, the inhibitory neurons in TPJ and mPFC receive other-relevant information from STS or ACC, respectively. The information of inhibitory neurons in TPJ is identical with the information of other-relevant stimuli in pSTS, and the information of inhibitory neurons in mPFC is identical with the information of other's belief in dmPFC. The temporary neurons in TPJ receive self-relevant information from STS, and the temporary neurons' information is identical with the information of self-relevant stimuli in IPL. The self-perspective inhibition takes place in TPJ and the self-belief inhibition takes place in mPFC. Then we test the effect of these connections in the false-belief task, and observe that the different maturation of connections leads to different permanence in the task. In this figure, the inhibitory neurons (In) are used to inhibit information in IPL or vmPFC, and the inhibitory result (InR) is the result of their interaction. If the connections between IFG and TPJ, IFG and vmPFC are mature, the activated neurons of InR will activate IFG to inhibit the information in InR neurons, and then make the correct information as the output of TPJ and mPFC. These connections will not influence the process of reasoning about self-belief.

## 5. Discussion

In this section, we will discuss the characteristics of the model, the reasons why robot experiments and cognitive experiments are not completely consistent, and the possible mechanisms of why toddlers fail in high inhibition tasks.

Compared to the previous models which we introduce in the related works, our model explores and is fundamentally based on the role of self-experience. In our model, robots learn to understand object permanence and visual access of blindfold or turn around from self-experience, then use it to infer other's belief and predict their actions. All of the participant robots learn the ability of understanding object permanence from the same experience. In the Opaque-and-Transparent Blindfold Test, they are divided into opaque blindfold group and transparent blindfold group. Even though the visual inputs of both groups in the test stage are identical, the different experience with an opaque blindfold or transparent blindfold leads to different performances. In Turn Around Test, the different behaviors of the actor robot in the test stage result in different performances. Compared with the recently published work from Patacchiola and Cangelosi (2020): (1) Our model is based on spiking neural networks, and just uses the STDP to successfully reproduce the complex cognitive function of ToM, hence more biological plausible. And their model is based on an actor-critic (AC) framework, an epigenetic robotic architecture (ERA) and a Bayesian network (BN). (2) Our model considered more brain regions that have been consistently found in many experimental paradigms of ToM, such as the TPJ that used for self-other distinction, the IFG that used for self-perspective inhibition and self-belief inhibition, etc. (3) Our model is used for the false belief task, which is one of the most classical and widely used experimental paradigms of ToM, and their model challenges a different task. The two studies have complementary contributions to the ToM models through bio-inspired mechanisms.

Through the integration of biological inspirations and computational modeling, we suggest that the self-experience, maturation of correlated brain areas (e.g., calculation capability) and connection between brain areas (e.g., inhibitory control) will have great influence on the participant's performance in the false-belief task.

As indicated in Scott and Baillargeon (2017), the false belief tasks contain spontaneous-response and elicited-response tasks that belong to the implicit task and explicit task, respectively. The difference in spontaneous-response and elicited-response tasks is that the former investigates the capacity of false belief understanding by spontaneous behavior such as anticipatory-looking, preferential-looking, etc with a non-verbal task, and the latter investigates this capacity by answering direct questions that predict agent's behavior who has a false belief with the verbal task. Children can pass the spontaneous-response task before 2 years old, but they can not pass the elicited-response tasks until about 4 years old. Our tasks on robots are not completely consisted with Senju et al. (2011) on 18-month-olds infants and Southgate et al. (2007) on 2-year-olds infants. Both of them used spontaneous-response to test the infants on whether they can pass the task. And in the test trial, they removed the object from the scene to make infants pass the task easier. In our task, we determine whether the robot can pass the task by detecting the direction of the finger which makes the results more intuitive. And in the test trial, we move the object to the other box. Setoh et al. (2016) found that 2.5-year-olds toddlers could succeeded in a traditional false-belief task with reduced processing demands. Toddlers could pass the elicited-intervention and low inhibition task (removing object from the scene) which is described by language and picture, but would fail in high inhibition task (moving the object to another box). They thought the reason why toddlers failed in high inhibition task is that toddlers cannot inhibit the response of the actual location of the object. We suppose that the core mechanism of belief reasoning is identical in both tasks, and the only difference should be in the process of motor response. In the motor response process of the elicited-intervention task, it may use the brain areas which control the hand movement. And in spontaneous-response task, it may use the brain areas which control the eye movement. The main reason why we use high inhibition task to replace low inhibition task is that the behavior of the robots in the true belief task is more intuitive to be understood (for the high inhibition task, the robot can point to the position of the object, while for the low inhibition task, the objects are removed outside the scene, and the robot cannot point to their positions).

And we suppose that the reason why toddlers failed in high inhibition task should be related to the lack of motor response ability rather than ToM (e.g., understanding that others have beliefs that are different from one's own). As shown in Figure 9, the connection between IFG and TPJ are matured but the connection between IFG and vmPFC are not, both of the two candidate responses are activated when the participant robot is asked “Where is the ladybird according to the blue robot.” The participant robot will tend to select action directed by self-belief rather than randomly, as the result of behavior data shown in Setoh et al. (2016) and Samson et al. (2005). In the low inhibition task, the participant has no idea about the object's location and one of the two candidate motor responses are activated, so the participant robot can succeed in this task. And in Samson et al. (2005), the participant who has a lesion of the right inferior and middle frontal gyri performed well in low-inhibition false-belief task but failed in the high-inhibition task.

Here we don't attempt to compare our model with the traditional Theories of ToM as Theory Theory (Gopnik and Wellman, 1994), Simulation Theory (Gallese, 1998), etc., because what we focus on in this paper is to build a computationally feasible model which could uncover the detailed mechanisms of ToM and enhance our understanding of how the self-experience, maturation of correlated brain areas and connection between brain areas affect the participant's performance in the false-belief task.

## 6. Conclusion

The computational model for the robotic ToM is regarded as one of the Grand Challenges for Artificial Intelligence and Robotics (Yang et al., 2018). Here we proposed a Brain-ToM model based on existing biological findings of ToM, and this model shows its relevance to ToM of human from the mechanism and behavior perspectives.

In summary, we propose a Brain-ToM model to enable machines to acquire the ability of ToM through learning and inferring based on self-experience. We validate the model by deploying it on humanoid robots. Our model successfully enabled the robot to pass the false-belief task, which is a classical task designed to understand the nature and mechanisms of ToM from Cognitive Psychology. The model and its application on robots show that current understanding on the mechanisms of the ToM can be computationally unified into a consistent framework and enable the robots to be equipped with the initial cognitive ability of ToM.

## Data Availability Statement

The original contributions presented in the study are included in the article/Supplementary Material, further inquiries can be directed to the corresponding author/s.

## Author Contributions

YZe conceived the initial idea. YZh and YZe designed the model, carried out, and analyzed the experiments. TZ, YZe, and DZ designed and implemented the SNN algorithm. FZ contributed to the inhibitory control mechanism. YZe, YZh, TZ, and EL wrote the manuscript.

## Conflict of Interest

The authors declare that the research was conducted in the absence of any commercial or financial relationships that could be construed as a potential conflict of interest.

## Supplementary Material

Movie S1 Visual access learning of blindfold from self-experience (opaque blindfold group). This process enables the participant robot understand that other robots wearing a blindfold cannot see.

Movie S2 Visual access learning of blindfold from self-experience (transparent blindfold group). This process enables the participant robot understand that other robots wearing a blindfold can see.

Movie S3 Familiarization phase. This process enables the participant robot understand the goal of the actor robot.

Movie S4 Opaque-and-transparent blindfold test (opaque blindfold group). The details in opaque blindfold group of blindfold test.

Movie S5 Opaque-and-transparent blindfold test (transparent blindfold group). The details in transparent blindfold group of blindfold test.

Movie S6 Turn around test (false-belief task). The details in false-belief task of turn around test.

Movie S7 Turn around test (true belief task). The details in true belief task of turn around test.

## Abbreviations

ToM: theory of mind
STS: superior temporal sulcus
TPJ: temporo-parietal junction
IPL: inferior parietal lobule
pSTS: posterior superior temporal sulcus
PCC: posterior cingulate cortex
ACC: anterior cingulate cortex
mPFC: medial prefrontal cortex
vmPFC: ventral medial prefrontal cortex
dmPFC: dorsal medial prefrontal cortex
IFG: inferior frontal gyrus
vPMC: ventral premotor cortex
M1: primary motor cortex.
